# RNA-Seq analysis revealed genes associated with drought stress response in kabuli chickpea (*Cicer arietinum* L.)

**DOI:** 10.1371/journal.pone.0199774

**Published:** 2018-06-28

**Authors:** Keyvan Mahdavi Mashaki, Vanika Garg, Ali Asghar Nasrollahnezhad Ghomi, Himabindu Kudapa, Annapurna Chitikineni, Khalil Zaynali Nezhad, Ahad Yamchi, Hasan Soltanloo, Rajeev Kumar Varshney, Mahendar Thudi

**Affiliations:** 1 Department of Plant Breeding and Biotechnology, Gorgan University of Agricultural Sciences and Natural Resources, Gorgan, Iran; 2 Center of Excellence in Genomics and Systems Biology, International Crops Research Institute for the Semi-Arid Tropics (ICRISAT), Hyderabad, Telangana, India; National Institute of Plant Genome Research, INDIA

## Abstract

Drought is the most important constraint that effects chickpea production globally. RNA-Seq has great potential to dissect the molecular mechanisms of tolerance to environmental stresses. Transcriptome profiles in roots and shoots of two contrasting Iranian kabuli chickpea genotypes (Bivanij and Hashem) were investigated under water-limited conditions at early flowering stage using RNA-Seq approach. A total of 4,572 differentially expressed genes (DEGs) were identified. Of these, 261 and 169 drought stress responsive genes were identified in the shoots and the roots, respectively, and 17 genes were common in the shoots and the roots. Gene Ontology (GO) analysis revealed several sub-categories related to the stress, including response to stress, defense response and response to stimulus in the tolerant genotype Bivanij as compared to the sensitive genotype Hashem under drought stress. In addition, several Transcription factors (TFs) were identified in major metabolic pathways such as, ABA, proline and flavonoid biosynthesis. Furthermore, a number of the DEGs were observed in “*QTL-hotspot*” regions which were reported earlier in chickpea. Drought tolerance dissection in the genotypes revealed that the genes and the pathways involved in shoots of Bivanij were the most important factor to make a difference between the genotypes for drought tolerance. The identified TFs in the experiment, particularly those which were up-regulated in shoots of Bivanij during drought stress, were potential candidates for enhancing tolerance to drought.

## Introduction

Chickpea (*Cicer arietinum* L.) is the second most important cool season grain legume crop cultivated on 13.98 M ha globally in 59 countries with a total production of 13.73 M tons [1]. It is a self-pollinated crop with a basic chromosome number eight (2*n* = 2*x* = 16) and genome size of ~738 Mb [2]. It is cultivated on residual soil moisture by resource poor farmers especially in the arid and semi-arid regions of South Asia and Sub-Saharan Africa. Major chickpea producing countries include India, Australia, Turkey, Myanmar, Pakistan, Ethiopia, Iran, etc. In terms of chickpea production Iran ranks seventh, while productivity (500 kg/ha) is much less than the world’s average productivity. Desi (light to dark brown in color) and kabuli (white or beige colored seed) are two types of chickpea cultivated globally. Like other legumes, chickpea enhances the soil fertility by fixing atmospheric nitrogen and also serves as a valuable source of proteins, vitamins and essential amino acids [3]. The productivity of chickpea has been low due to several abiotic and biotic stresses.

Among abiotic stresses, drought alone leads to >50% annual yield losses in chickpea. End season drought or terminal drought delays flowering and reduces growth duration particularly at reproductive stage and finally leads to yield losses [4–5]. Considering drastic effects of drought at reproductive stages, identification of drought tolerance genes during flowering is essential for understanding molecular mechanisms of tolerance and plant adaptation. Advances in genomics research during last decade facilitated development of several molecular markers [6], genetic maps [7] which increased our understanding of the genetics of complex traits like drought. Several approaches like linkage mapping [8] and genome-wide association mapping [9] were employed in past to dissect drought tolerance mechanism in chickpea. Further, efforts were also made to fine map this genomic region to few kilobases [10–11]. Unprecedented developments in next generation sequencing (NGS) technologies not only decoded the genome architecture of several important crop plants [12–13] but also provided insights into the proteome and metabolome of model plant species [14]. In the case of chickpea using NGS technologies, draft genomes of both desi and kabuli chickpea have been deciphered [2,15]. In addition, whole genome resequencing (WGRS) of parental lines of mapping populations provided large scale single nucleotide polymorphisms (SNPs) data for trait improvement [16]. Insights into the temporal and spatial trends in diversity of released varieties have been reported using WGRS approach [17]. Although large amount of transcriptome data on desi genotypes were generated in recent years [18–22], specific role of stress responsive genes and their interactions are still unknown in kabuli chickpea genotypes.

RNA-Seq approach, that uses deep sequencing technologies, is a cost effective way for transcriptome studies [23]. Furthermore, RNA-Seq has been the technology of choice for identification of novel genes and isoforms, detection of variants including expressed SNPs, InDels, SSRs and gene fusion events [24]. RNA-Seq approach enabled a clear understanding of differentially expressed genes and physiological responses under drought stress in sorghum [25]. In case of barley, genes responsible for reproductive success under drought stress were reported [26]. High temperature and drought stress-related genes were reported in case of *Brassica juncea* [27].

In the present study, RNA-Seq was employed to investigate transcriptome profiles in drought-responsive contrasting genotypes of Iranian kabuli chickpea under drought stress in root and shoot tissues at early flowering stage. RNA-Seq on Illumina platform provided a thorough scenario on the whole chickpea transcriptome in response to drought stress. Several categories of key genes involved in drought response have been identified.

## Materials and methods

### Plant Material, growth conditions and drought stress treatment

Two contrasting Iranian chickpea kabuli genotypes Bivanij (drought tolerant) and Hashem (drought sensitive) were used in this study. The seeds were obtained from Sararood Rainfed Agriculture Research Station, Kermanshah, Iran. Two seeds were sown in pots, (0.21 m deep with 0.25 m diameter) containing 9 kg of vertisol soil and fertilized with sterilized farm yard manure as one part for 20 parts soil (v/v), in a greenhouse (ICRISAT, Hyderabad, India). The plants were thinned to one healthy plant per pot after 8 days of germination. Five biological replicates of each genotype and treatment were maintained under glasshouse conditions. The temperature inside the greenhouse were at a maximum of 24–28°C and a minimum of 12–22°C, and a day-time relative humidity of 30–70% with a maximum solar radiation incidence of >1200 μE m^−2^ s^−1^. The plants were grown under well-watered conditions for 4 weeks before initiating a dry-down experiment. One day before imposing stress, all the pots were saturated with water and allowed to drain excess water in 24 hours to maintain the field capacity so that the soil moisture amount in each pot were uniform. Then each pot was weighed to know the amount of water at field capacity. All pots were weighted every day on a sensitive balance with a resolution of plus or minus 0.1 g. The control plants were then watered to restore the soil to about 80% of field capacity by adding a pre-calculated amount of water while the stress plants were remained stressed for 15 days until the stress level reached to 20% of their field capacity. All the root and the shoot tissue samples were harvested in at least three biological replicates. The tissues were washed thoroughly with 0.1% DEPC water, frozen in liquid nitrogen and stored at -80°C until RNA extraction.

### RNA extraction, Illumina sequencing and data quality control

Total RNA was extracted from 24 samples (two genotypes × two treatments × two tissues × three biological replicates) using NucleoSpin RNA Plant kit (MACHEREY-NAGEL GmbH & Co. KG, Germany), according to manufacturer’s instructions. Quantity and quality of the RNA was determined using Nanodrop 8000 Spectrophotometer (ThemoScientific, USA) and Agilent 2100 Bioanalyzer (Agilent Technologies, USA). The RNA (with RIN ≥8) was pooled from the three biological replicates of each sample and then a total of eight samples (namely Bivanij root drought control (BRDC), Bivanij shoot drought control (BSDC), Bivanij root drought stress (BRDS), Bivanij shoot drought stress (BSDS), Hashem root drought control (HRDC), Hashem shoot drought control (HSDC), Hashem root drought stress (HRDS) and Hashem shoot drought stress (HSDS) were processed further using Illumina TrueSeq RNA Sample Prep kit for cDNA library preparation. In brief, it includes purification of the polyadenylated RNA, the RNA fragmentation, cDNA synthesis and adaptor and barcode ligation. After cluster generation using Illumina cBot, the libraries were sequenced on Illumina HiSeq 2500 platform to generate >30 million 125 bp length paired-end (PE) reads for each sample. The high-quality reads were obtained after several steps of quality checks which included trimming, removal of adaptor/primer and low quality reads using Trimmomatic v 0.35 [28] and NGS-QCbox [29].

### Reference-based mapping and assembly

The transcriptome data was analyzed using Tuxedo pipeline [30]. The filtered high-quality reads of all samples were mapped on kabuli chickpea reference genome (v1.0) using TopHat v2.1.0 [31] with default parameters. The mapped reads from each sample along with the genome GFF were used to perform reference annotation based transcript (RABT) assembly using Cufflinks v2.0.2 [32,33]. These Cufflink assemblies were then compared and merged using Cuffmerge to remove transfags and generate a consensus assembly for downstream analysis [32].

### Identification of differentially expressed genes (DEGs), GO and pathway analysis

The normalized expression of all the samples was estimated in FPKM (fragments per kilobase of exon per million fragments mapped). The changes in the relative abundance of the genes between the different genotypes/treatments/tissues were estimated usingCuffdiff [34]. The genes exhibiting significant differences (at least two-fold change and a FPKM of > = 3 for either of the sample) were considered to be differentially expressed genes. Further, CummeRbund was used to plot and visualize abundance and differential expression results from Cuffdiff. The heatmaps showing the expression profiles were generated using MultiExperiment Viewer v4.9 [35]. To understand the functional classification of DEGs, the DEGS were subjected to BLASTX similarity search against NCBI non-redundant protein database with an E-value cut off of ≤10^−5^ followed by their annotation using Blast2go [36]. Pathway analysis of the DEGs was carried out using KEGG database. For identification of transcription factor encoding genes, the DEGs were searched against Plant transcription factor database (PlantTFDB 4.0) with an E-value cut off of ≤10^−5^.

### Quantitative real-time PCR (qRT-PCR) analysis

To validate RNA-Seq results, 12 genes (S1 Table) selected from the DEGs analysis were subjected to quantitative real-time PCR (qRT-PCR). The primer pairs were designed using Primer Express (v3.0) software (Applied Biosystems, Foster City, CA). The RNA samples used for sequencing (mentioned above) from three biological replicates were used for the validation. The total RNA was reverse-transcribed using SuperScript III First-Strand synthesis kit (Invitrogen, life technologies, USA). After quality control by Nanodrop spectrophotometer, the cDNA samples were diluted and normalized with the house keeping gene Glyceraldehyde 3-phosphate dehydrogenase (*GAPDH*). The qRT-PCR analysis was performed using Applied Biosystems 7500 Real-Time PCR System with SYBR green chemistry (Applied Biosystems, USA) in two technical replicates. In brief, PCR reactions were carried out in 10 μl reaction volume containing 30 ng of first strand cDNA, 1X PCR buffer, 125 mM dNTPs, 1.5 mM MgCl_2_, 0.2 mM primers and 1U *Taq* polymerase and programed as follows: 50°C for 2 min and denaturation at 95°C for 10 min followed by 40 cycles of denaturation at 95°C for 15 sec and annealing and extension at 60°C for 1 min. The data obtained from different PCR runs or cDNA samples was analyzed using the mean of the CT values of the three biological replicates that were normalized to the mean CT values of the house keeping gene *GAPDH*. The expression values were calculated using the 2^_ΔΔCt^ method, student's t-test was used to calculate significance and relative transcription levels are presented graphically [37].

## Results

### High throughput sequencing and assembly

A total of 642.93 million reads were generated from the transcriptome sequencing of eight samples with about 60.8 to 94.8 million reads per sample. The reads with adapter contamination and low base quality (≤ Q20) were removed using Trimmomatic and NGS-QCbox [29]. As a result, in total 593.94 million (92.38%) high quality reads were obtained. A total of 568.8 million reads (95.76%) were mapped on to chickpea reference genome (v1.0) [2] using TopHat2 software. On average, 71.1 million reads (95.7%) were mapped to the reference genome of chickpea for each sample (Table 1).

**Table 1:** Summary of Illumina sequencing data and mapped reads for the samples.

Genotype	Tissue	Treatment	Sample ID	Total reads	Mapped reads	Mapping rate (%)	Unique match	Multi-position match
Bivanij	Root	Control	BRDC	91,293,742	80,263,790	95.4	77,898,377	23,654,13
		Drought stress	BRDS	71,897,392	63,330,795	95.3	61,694,984	16,358,11
	Shoot	Control	BSDC	84,473,708	75,724,423	96.0	73,832,159	18,922,64
		Drought stress	BSDS	94,898,074	83,051,032	95.1	80,903,347	21,476,85
Hashem	Root	Control	HRDC	79,714,416	70,462,846	95.8	68,448,980	20,138,66
		Drought stress	HRDS	60,810,664	53,408,459	95.8	52,031,036	13,774,23
	Shoot	Control	HSDC	84,992,750	76,347,429	96.2	74,519,352	18,280,77
		Drought stress	HSDS	74,858,446	66,211,881	96.7	32,458,029	33,753,852
Total				642,939,192	568,800,655			
Average				80,367,399	71,100,081.80	95.7		

### Global gene expression analysis

**Fig 1:**
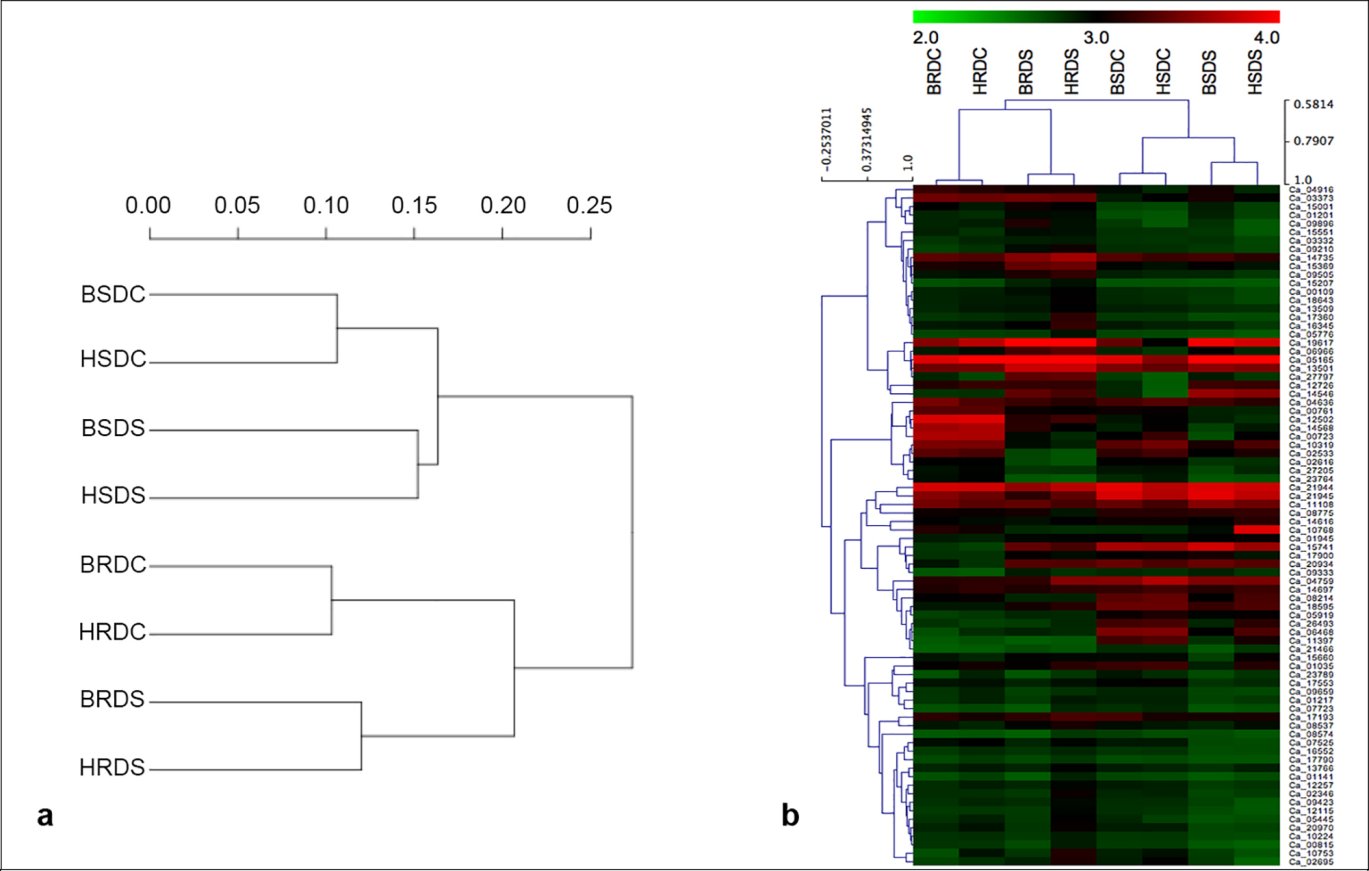
Correlation of the expression values of the samples. (A) Dendogram depicting correlation among different samples based on global expression profiles. (B) Heatmap grouped the eight samples based on log_10_ transformed FPKM (>400) of the highly expressed genes. Color scale shows high and low expressions as red and green, respectively.

A gene was considered to be expressed in a sample if its FPKM ≥1. A total of 22,363 genes were expressed in at least one of the eight samples analyzed. Among these, 7,589 genes were found to be constitutively expressed in all samples with coefficient of variation less than 10%. The global expression profiles of all samples grouped root and shoot samples separately in accordance to their phenotypic drought tolerance/sensitive expression. In roots, two control and two drought stress samples were grouped together, whereas the correlation between the drought stress samples was less than the control samples in the shoots (Fig 1A). The eight samples were categorized based on highly expressed genes. The expression values of 80 highly expressed genes (with FPKM value >400) among the samples were used to develop the heatmap (Fig 1B). For better visualization of the heatmap, log_10_ transformed FPKM for the genes in all the samples were calculated. The 80 highly expressed genes were placed in two large clusters containing 25 and 55 members for individual cluster. The expression profiles were different between the clusters. Many sub-clusters were located per cluster based on the difference among the genotypes, the tissues and the treatments.

### Differentially expressed genes (DEGs)

**Fig 2:**
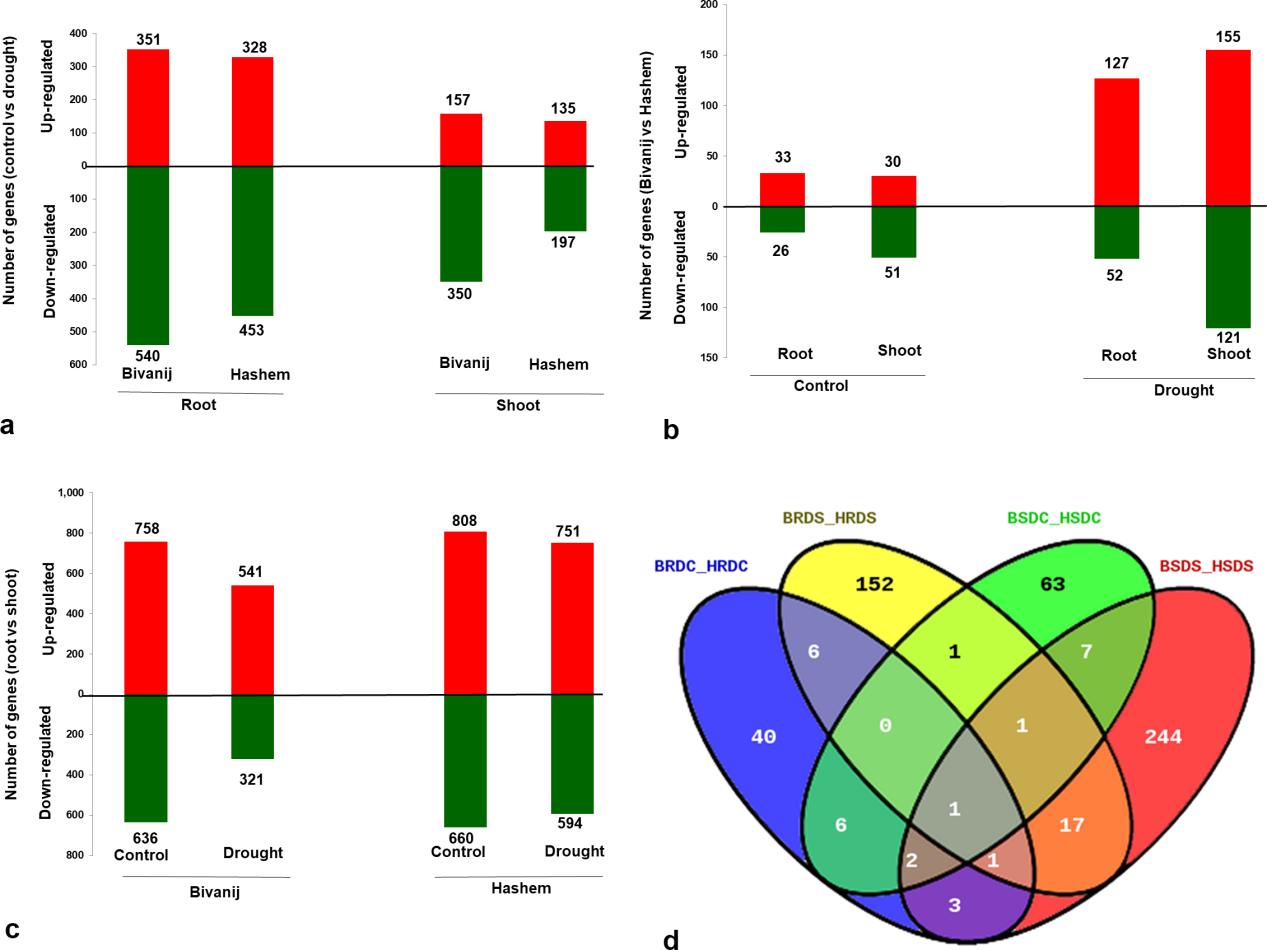
Number of differentially expressed genes. Comparison between: (A) The treatments (B) The genotypes and (C) The tissues. (D) Venn diagram shows the overlapped genes by comparing the genotypes under the control and the drought treatments in the roots and the shoots.

A total of 4,572 differentially expressed genes (DEGs) were identified by comparing the eight samples using Cuffdiff, of which 428 (9.37%) were novel. Of the 4,572, 94.1% (4,302) DEGs mapped uniquely and 5.9% (270) DEGs mapped to multiple locations in the genome. The highest number of DEGs (1,468) were identified between the roots and the shoots of Hashem under the control condition (HRDC vs HSDC) and the lowest number of DEGs (59) were in comparison of the roots of Bivanij and Hashem under the control condition (BRDC vs HRDC) (S1 Fig). For better understanding, differential gene expression analysis was performed based on the tissues, the treatments and the genotypes. Under the drought stress, DEGs in the roots of Bivanij were higher as compared to Hashem (891 vs 781) and were similar with DEGs in the shoots (507 vs 332). Overall, the number of down-regulated genes under drought stress conditions were higher as compared to the control conditions (Fig 2A). In addition, higher number of genes were differentially expressed between the tolerant and the sensitive genotypes in the drought stress as compared to the control condition. In both the control and the drought stress conditions, DEGs were more in the shoots than the roots. Under the drought stress, 127 and 52 genes were up-regulated in roots of Hashem and Bivanij, respectively. While, 155 and 121 genes were up-regulated in the shoots of Hashem and Bivanij, respectively (Fig 2B). Finally, DEGs between the tissues in two genotypes under both the control and the drought stress conditions were compared. As shown, the DEGs between the roots and the shoots in the sensitive genotype were more than those in tolerant genotype, and in both the genotypes, the DEGs were reduced during the drought stress as compared to the control condition. Overall, in all comparisons, number of the up-regulated genes were more than down-regulated genes (Fig 2C**)**. In addition, overlapped differentially expressed genes between Bivanij and Hashem under the control and the drought stress conditions were analyzed (Fig 2D). In comparison the blue and the orange ovals, drought-responsive genes were identified, which were specifically expressed in the roots, including 171 (121 up-regulated in Hashem and 50 up-regulated in Bivanij), 51 (28 up-regulated in Hashem and 23 up-regulated in Bivanij) and eight drought-responsive, control and common genes, respectively. The other comparison was performed between green and red ovals in order to identify drought responsive genes, which were specifically expressed in the shoots. The results exhibited that 265 genes (153 up-regulated in Hashem and 112 up-regulated in Bivanij) were specifically expressed under the drought stress, 70 genes (28 up-regulated in Hashem and 42 up-regulated in Bivanij) were expressed under the control condition and 11 genes were common. Finally, 261, 169 and 17 drought responsive genes were expressed in the shoots, the roots and common genes between the tissues, respectively (S2 Table and S3 Table). Interestingly, only one gene (Ca_02523), an integral component of membrane and transporter activity was showing an overlap in all combinations.

### Functional classification of DEGs

**Fig 3:**
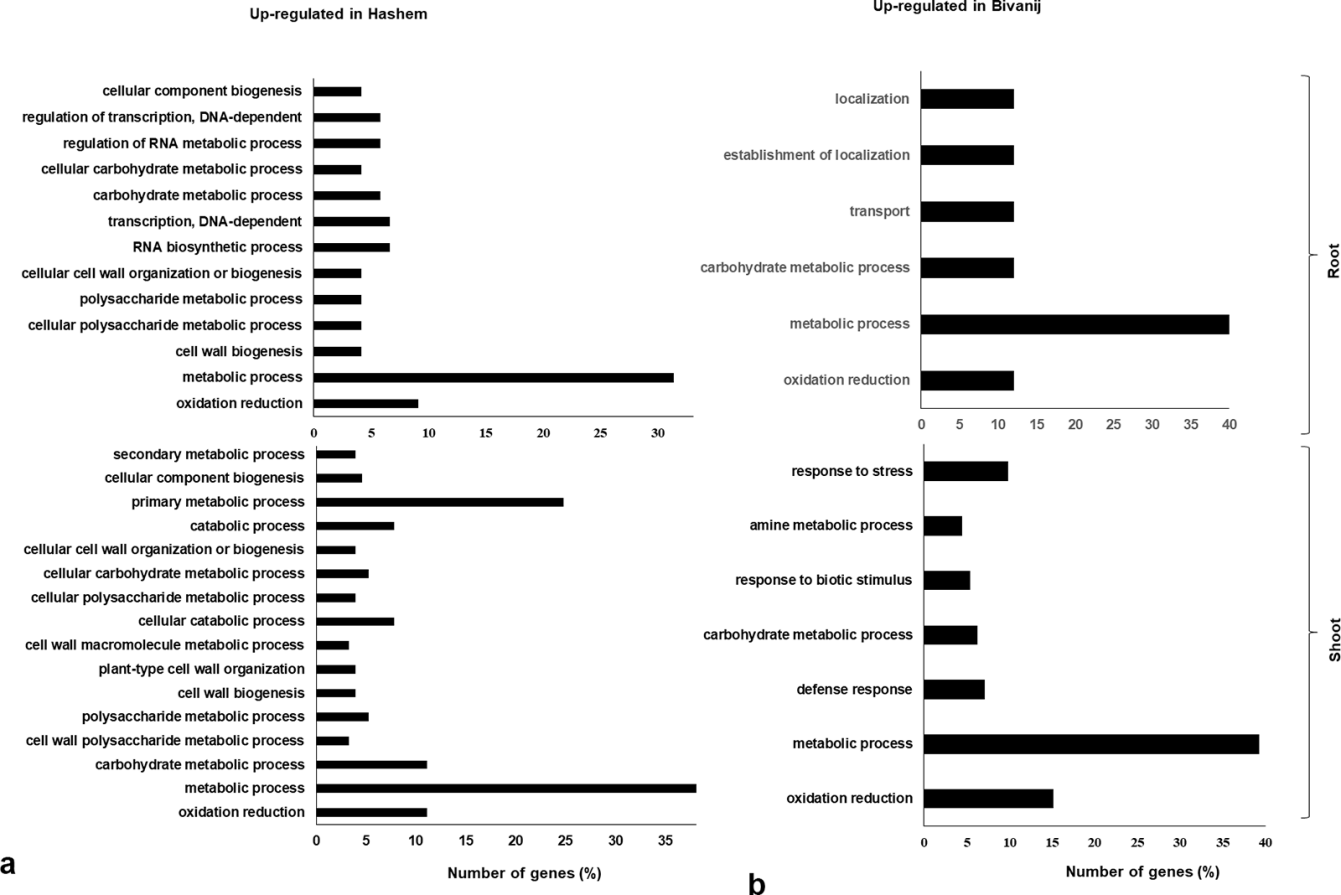
GO enrichment analysis of DEGs identified between the tolerant and the sensitive genotypes under the drought stress in the roots and the shoots.

Gene ontology (GO) analysis was performed to functionally categorize the 4,572 differentially expressed genes into three principal categories: cellular component, molecular function, and biological process (S2 Fig). In cellular component category, cell and cell part sub-categories were found to be mostly enriched and in molecular function category, binding, catalytic activity followed by transporter and transcription regulator were the most enriched categories. A large number of GO sub-categories, such as metabolic and cellular process, biological regulation, pigmentation, localization and response to stimulus were found to be significantly enriched in biological process category. GO analysis was also performed specifically for the drought responsive genes in the category of biological process (Fig 3). The sub-category, metabolic process with highest number of genes as well as oxidation-reduction were commonly found in all the conditions, while other biological processes were differently enriched. In the roots, several GO sub-categories associated with RNA transcription and cell wall were involved in Hashem, whereas localization, transport and carbohydrate metabolic processes were found in Bivanij. In addition to metabolic process and oxidation-reduction, primary metabolic process was highly enriched in shoots of Hashem, followed by catabolic, cellular and carbohydrate metabolic processes. Although a higher number of enriched sub-categories were in Hashem in both roots and shoots, however a number of major sub-categories, including response to stress, defense response and response to biotic stimulus were significantly and specifically enriched in the shoots of the tolerant genotype, indicating the vital role of Bivanij for plant drought tolerance.

### Differential expression of genes associated with KEGG metabolic pathways

**Fig 4:**
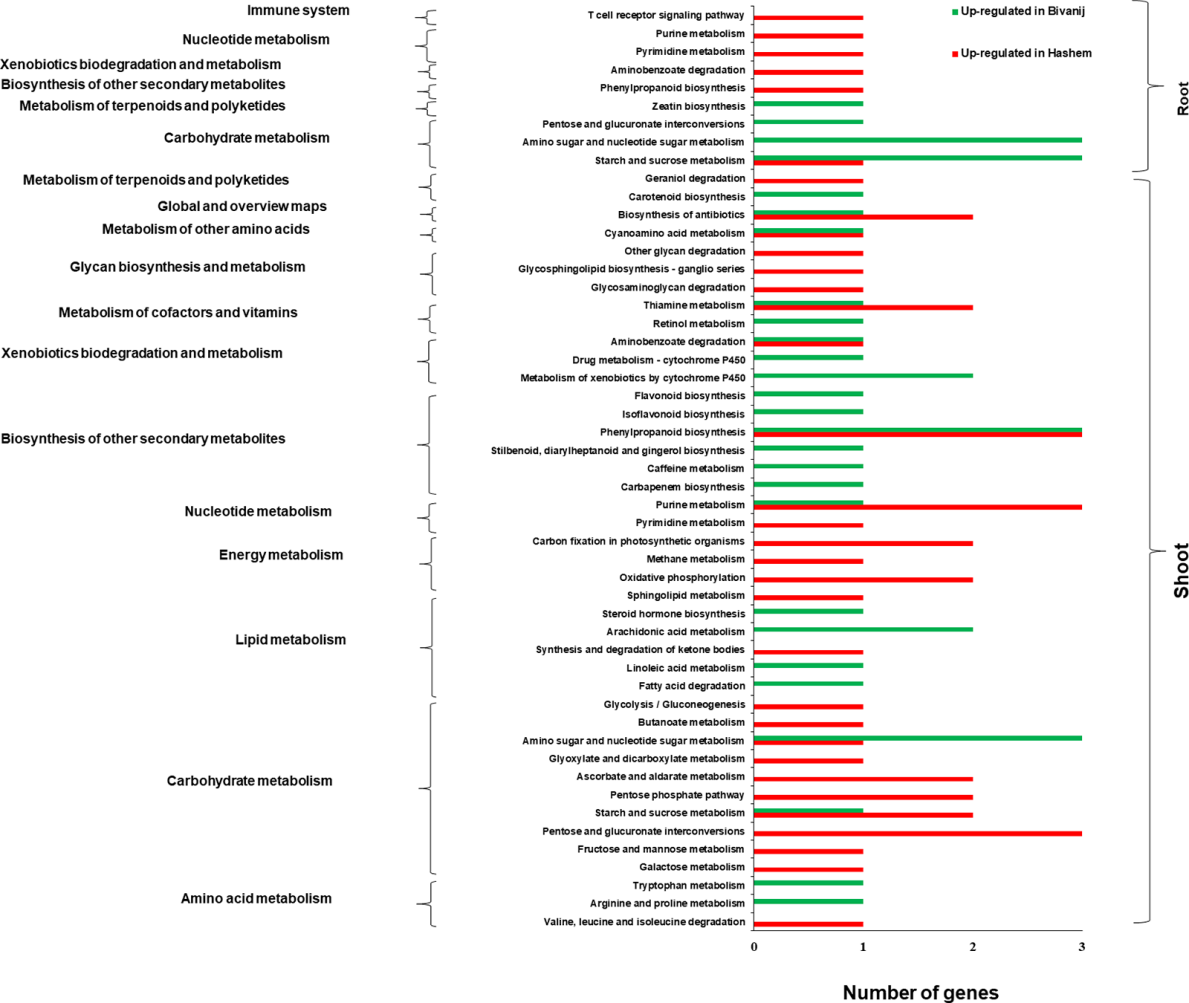
KEGG significantly metabolic pathways in the roots and the shoots obtained from comparing the genotypes under the drought stress.

KEGG pathway analysis was performed in order to compare the metabolic pathways between Bivanij and Hashem genotypes under the drought stress in the roots and the shoots (Fig 4). The enzymes with most frequency were monooxygenase (13.3%), aldolase (8%) and lactase (6%) in the shoots and adenylyl transferase (10%) and RNA polymerase (10%) in the roots. Totally, the results of KEGG pathway enrichment were in agreement with GO analysis. Various pathways of carbohydrate metabolism, biosynthesis of other secondary metabolites, energy metabolism, xenobiotics biodegradation and metabolism and nucleotide metabolism were enriched in the shoots with the highest number of the genes, whereas the pathways associated with carbohydrate metabolism were frequent in the roots.

### Differentially expressed transcription factors (TFs)

**Fig 5:**
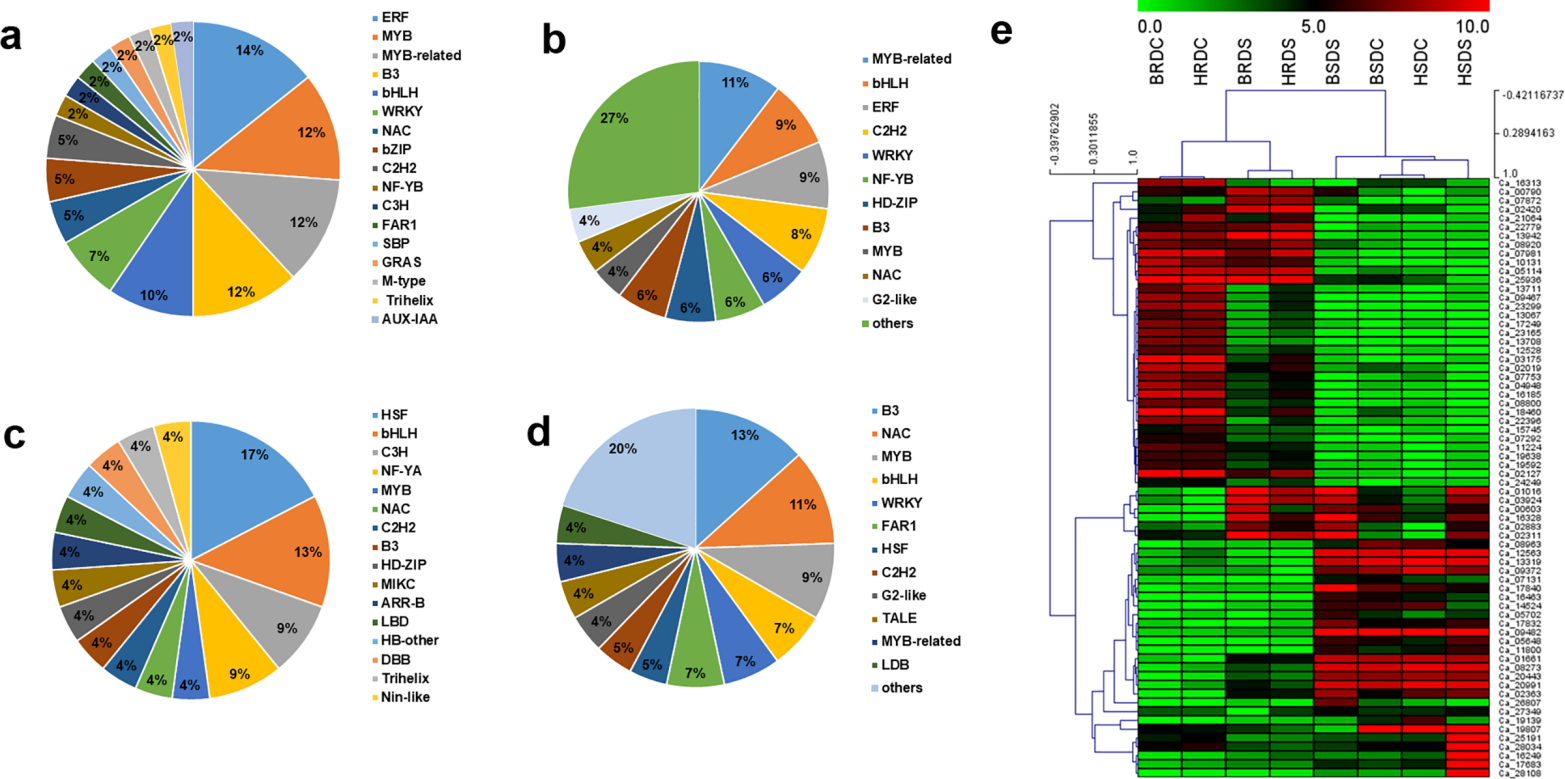
Distribution of TF families in comparison between the genotypes under the drought stress. (A) Up-regulated in the roots of Hashem (B) Up-regulated in the shoots of Hashem (C) Up-regulated in the roots of Bivanij and (D) Up-regulated in the shoots of Bivanij. (E) Heatmap showing expression profile of NAC transcription factor family across different samples.

A total of 1,806 transcription factor related genes were identified from all the conditions and the top 20 TF families are shown in S3 Fig. The largest members of the TFs belonged to bHLH family (101), followed by ERF (87), kinase superfamily (76), NAC (74), MYB (72), WRKY (72), etc. The distribution of the differentially expressed TFs between Bivanij and Hashem under the drought stress was separately investigated in the roots and the shoots (Fig 5A–5E, S4 Table and S5 Table). Overall, 65 DEGs encoding TFs were found in the roots, including 42 (17 TF families) up-regulated in Hashem and 23 (16 TF families) up-regulated in Bivanij. In the shoots, 93 differentially expressed TFs were found between the genotypes during the stress condition, including 48 (24 TF families) up-regulated in Hashem and 45 (21 TF families) up-regulated in Bivanij. Six common differentially expressed TFs were also identified in the roots and the shoots. Interestingly, bHLH (Ca_09798) and MYB-related (Ca_19895) were induced in the roots of Hashem and repressed in the shoots. The distribution of the members of the differentially expressed TF families between the genotypes was different based on the tissue types and the genotypes. In the roots, the TF families, ERF (14%), MYB (12%), MYB-related (12%) and B3 (12%) were up-regulated in Hashem and HSF (17%), bHLH (13%), C3H (9%) and NF-YA (9%) were up-regulated in Bivanij with the highest members. However, in the shoots, the families MYB-related (11%), bHLH (9%), ERF (9%) and C2H2 (9%) with the high members were up-regulated in Hashem, whereas the members B3 (13%), NAC (11%) and MYB (9%) were up-regulated in Bivanij. Expression patterns of NAC transcription factor across different samples through heatmap which clearly indicated different expression level of NAC TF family in different tissues, treatments and genotypes.

### DEGs coincident with drought tolerance-related QTLs

In the present study, we found two (Ca_04551 and Ca_04552) and three (Ca_04561, Ca_04564 and Ca_04569) DEGs located in “*QTL-hotspot_a*” (of 15 genes) and “*QTL-hotspot_b*” (of 11 genes) regions, respectively, reported earlier by Kale et al. [11] (S4 Fig, S6 Table). No significant difference observed in the expression patterns of these five DEGs between the tolerant and the sensitive genotypes in the roots, however Ca_04551 and Ca_04564 were up-regulated in the shoots of Hashem. Ca_04569 was up-regulated only in the roots of Bivanij under drought stress and was annotated as ‘inositol polyphosphate-related phosphatase’. Ca_04551 was annotated as, ‘aldo/keto reductase’ and ‘potassium channel, voltage-dependent, beta subunit, KCNAB-related’. Ca_04552 was involved in ‘chlorophyll A-B binding protein’. The functions of Ca_04561 were associated with ‘E3 ubiquitin-protein ligase Msl2, zinc RING finger’. Finally, Ca_04564 was found as ‘leucine-rich repeat’. Among them, Ca_04551, Ca_04564 and Ca_04569 were TFs related to bHLH, S1Fa and M-type families, respectively.

### Validation of differentially expressed genes

In order to validate the sequencing results, quantitative real time PCR (qRT-PCR) analysis was performed using 12 drought stress responsive genes selected from the *in silico* analysis of the DEGs.

**Fig 6:**
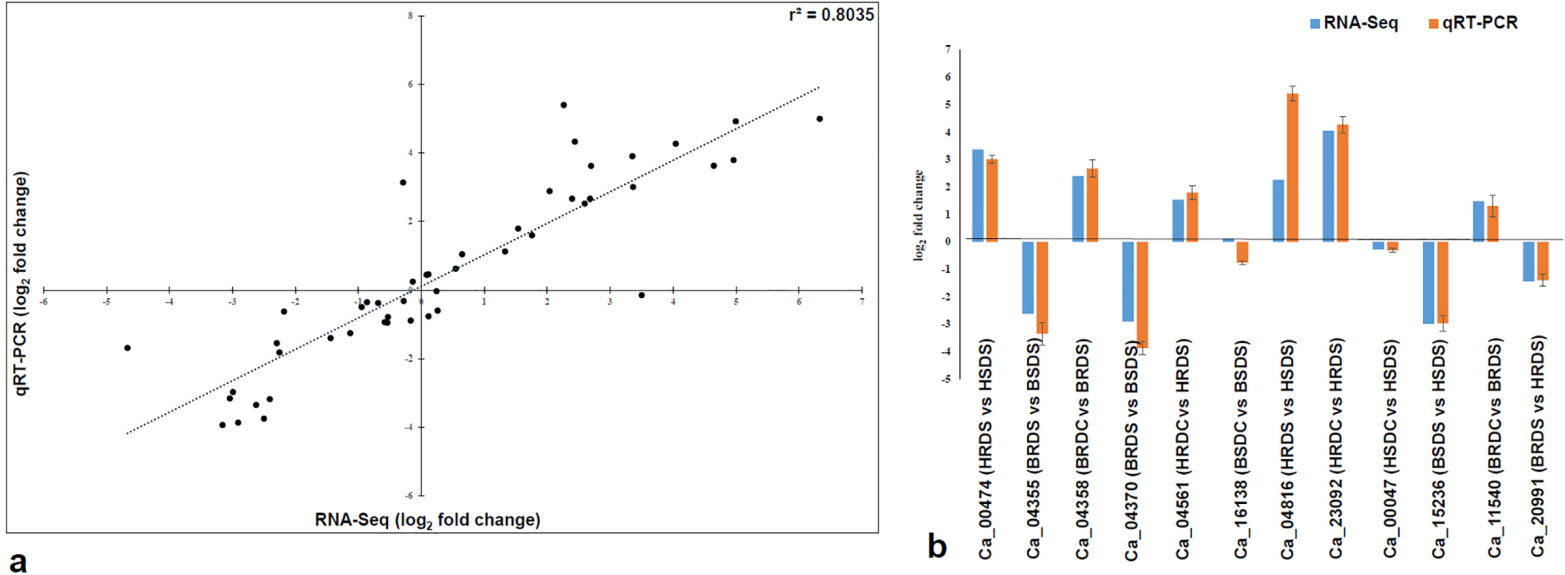
Validation of the RNA-Seq using the qRT-PCR. (A) Close correlation (r^2^ = 0.80) of 12 selected genes between two approaches. (B) Comparison of the RNA-Seq and the qRT-PCR considering the expression of the 12 selected genes in some combinations for instance.

Comparison of the expression values of the RNA-Seq and the qRT-PCR results under the control and the drought stress conditions indicated a strong and consistent correlation (r^2^ = 0.80) between the RNA-Seq and the qRT-PCR methods (Fig 6A). For instance, Ca_04358 and Ca_04561 showed up-regulation under drought stress in both the genotypes studied. On the other hand, two genes Ca_04355 and Ca_04561 showed down-regulation under drought stress conditions. These results are support *in silico* data. Further, the expression values of the 12 genes for RNA-Seq and qRT-PCR are shown as graphical representation (Fig 6B). Most of them revealed similar expression patterns. Interestingly, the expression of Ca_15236 as an important gene involved in the drought stress was almost same (-2.99 fold change in RNA-Seq and -2.97 fold change in qRT-PCR) between the two approaches.

## Discussion

Occurrence of drought during flowering time (early drought) and late at pod filling stage (terminal or end season drought) were reported to have significant impacts on chickpea yields. Drought being a complex trait, therefore understanding genetics of trait and genes associated with the drought tolerance has been the major area of focus in case of chickpea. The knowledge about these target drought responsive genes would enable development of drought tolerant cultivars through molecular breeding or transgenic approaches. Genomic regions/QTLs for drought tolerance have been reported using bi-parental mapping populations as well as diverse germplasm lines [38,8]. Attempts were also made to understand drought stress transcriptome [39,18,21,22], however these studies were on desi genotypes. In present study, drought tolerance was investigated in two Iranian kabuli chickpea genotypes Bivanij (tolerant) and Hashem (susceptible) at early flowering stage under drought stress conditions using RNA-Seq approach to understand the global transcriptome.

The GO enrichment analysis could functionally classify the DEGs and about 10% of the whole DEGs were assigned in response to stimulus. The most important stress-related GO terms such as response to stress, defense response and response to biotic stimulus were found to be specifically involved for up-regulated genes in the shoots of the tolerant genotype as compared to the sensitive genotype. Generally, the GO analysis suggested that the DEGs in the shoots were responsible for creating difference in drought tolerance between the genotypes. Transcriptome studies conducted in chickpea desi type have also noted the same GO terms in response to abiotic stresses [18,21,22].

Plant hormones regulate growth and development and are involved in various environmental stresses through signal transduction. Abscisic acid (ABA) is a key hormone in plants and its roles, such as seed dormancy and germination, modulation of roots architecture, senescence, stomata regulation and abiotic stress responses have been reviewed by Sah et al. [40]. ABA can also induce transcription factors and subsequent stress responsive genes, which confer tolerance to abiotic stresses, such as drought [41,42]. In this study, the gene (Ca_00449) involved in carotenoid biosynthesis pathway in shoots, which produces ABA was identified. This gene annotated as TF (FAR1 family) was up-regulated in Bivanij. The enzyme zeta-carotene desaturase (EC:1.3.5.6) was controlled by this gene and catalyzes the conversion of neurosporene to lycopene. Several ABA biosynthesis-deficient mutants have been shown to be sensitive to environmental stresses [43]. Overall, manipulation of ABA biosynthesis genes are important tools to enhance the tolerance to stresses. In addition, a number of ABA and ethylene responsive genes were differentially expressed between the two genotypes. Auxin and cytokinin hormone biosynthesis pathways were also identified to show differential regulation between the genotypes under the drought stress in the shoots and the roots, respectively. Both of them were up-regulated in Bivanij, indicating the successes of this genotype for the growth and the development during the drought stress.

Proline is a primary metabolite and accumulates as an osmotic adjustment during dehydration [44]. Further, proline protects macromolecules and enzymes under stress conditions and provide energy for growth and survival [45]. Arginine and proline metabolism is one of the proline biosynthesis pathways, which was identified in the present study. Remarkably, two enzymes were identified in proline biosynthesis, including glutamate 5-kinase (EC:2.7.2.11) and glutamate-5-semialdehyde dehydrogenase (EC:1.2.1.41) induced by a B3 transcription factor. This gene (Ca_24241) was up-regulated in the shoots of Bivanij as compared to Hashem under the drought stress treatment.

The genes of three pathways in energy metabolism, including photosynthesis, oxidative phosphorylation and methane metabolism were up-regulated in the shoots of Hashem. Considerably, this can be an adaptive response to reduce energy consumption in Bivanij under the drought condition. Increased levels of carbon fixation and photosynthesis due to open stomata led to reduction of osmotic pressure and thereby increasing the rate of transpiration in Hashem (as shown in Sade et al. [46]). Reduction of oxidative phosphorylation and photosynthesis have been reported in *Bombax ceiba* due to increased ABA, stomatal closure and increased level of proline under drought stress [47]. Soluble sugars, such as starch and sucrose are known to regulate osmotic pressure of plants [48,49]. In this study, the tolerant and the sensitive genotypes exhibited different responses in the shoots and the roots during starch and sucrose metabolism. In the shoots, two genes were up-regulated in Hashem and one gene was up-regulated in Bivanij, whereas in the roots, three genes were induced in Bivanij and only one gene was induced in Hashem. Increased amount of soluble sugars can be involved in growth and development or osmotic adjustment and enhancing stress tolerance [50,51].

Drought stress damages the photosynthetic pigments and results in reduction of chlorophyll content [52]. The KEGG pathway of porphyrin and chlorophyll metabolism conducts the biosynthesis of chlorophyll *a* and *b*. In the present study, there was no significant difference between two genotypes for the genes in that pathway, while the comparison of the tissues under drought stress identified several genes up-regulated in the shoots of both genotypes. It seems that increased degradation of chlorophyll content in the shoots as compared to the roots led to enhanced expression of the genes encoding the enzymes of chlorophyll biosynthesis pathway under drought stress.

The genes encoding biosynthesis pathways of secondary metabolites were mostly induced in Bivanij except the phenylpropanoid biosynthesis, which was also induced in Hashem. Flavonoids are a large group of secondary metabolites, which act as antioxidant and protect plants against various biotic and abiotic stresses [53]. In this study, shikimate O-hydroxy cinnamoyl transferase enzyme (EC:2.3.1.133) involved in flavonoid biosynthesis was induced by ARF transcription factor (auxin response factor). This gene (Ca_05702) was up-regulated in shoots of Bivanij as compared to Hashem under the drought stress. Overexpression of gene *AtMYB12* involved in flavonoid biosynthesis enhanced drought and salt tolerance through accumulation of flavonoids in transgenic Arabidopsis plants [54].

Reactive oxygen species (ROS) are generated in cells during stress conditions, and enzymes like catalase, superoxide dismutase and peroxidase play an important role in detoxifying these compounds [55]. Four genes involved in hydrogen peroxide catabolic process were identified in present study, three of them were up-regulated in Hashem and only one gene (Ca_04125) was up-regulated in Bivanij. These results indicated the complexity of tolerance to abiotic stresses, such as drought.

Plants respond to diverse stresses through perceiving the stresses by receptors or sensors at membrane level. Then the secondary messages, such as calcium ion, inositol phosphates, ROS, cyclic nucleotides and ABA are involved to transduce the signals into nucleus. Finally, the signals induce or repress various transcription factors (TFs) and consequently, many stress-related downstream genes are regulated through specific binding the TFs with their corresponding cis-elements in promoters [56,57]. In the present study, various TF families were identified, and their distribution and frequency were found to be greatly different, depending on the tissues, the treatments and the genotypes. 21 genes containing stress-related annotations were identified by comparing Hashem and Bivanij under the drought stress. Four and 17 differentially expressed genes were found in the roots and the shoots, respectively, suggesting the higher roles of the shoots to make different responses to drought stress between Hashem and Bivanij. Remarkably, 14 genes were up-regulated in Bivanij and only seven genes were up-regulated in Hashem, indicating the potential of Bivanij to be considered as the tolerant genotype (S7 Table). Moreover, the stress-related genes were involved in various stresses, suggesting a crosstalk among different stresses. Recently Nakashima et al. [58] confirmed presence of interactions between TFs and regulatory networks involved in diverse stress responses, including drought, cold and heat. Out of 21 genes with stress-related annotations, nine genes (three in the roots and six in the shoots) were considered as TFs. The TFs, C3H (2), NAC (2) and MYB-related (1) were up-regulated in Bivanij under the drought stress, whereas M-type (2), MYB-related (1) and G2-like (1) were up-regulated in Hashem.

The gene families such as AP2/ERF and heat shock protein (HSP) are crucial protein factors involved in many developmental processes and stress responses. HSP90 as a highly conserved chaperone protein assists to refold, stabilize and maintain other proteins, which were damaged under stress conditions [59,60]. Agarwal et al. [61] dissected AP2/ERF and HSP90 genes families in chickpea, pigeonpea and common bean under various biotic and abiotic stresses. Interestingly, four genes from AP2/ERF (Ca_08430, Ca_09578, Ca_23799 and Ca_15031) and two genes from HSP90 (Ca_25811and Ca_25602) were found commonly with our differentially expressed genes; however, none of them were differentially expressed between Hashem and Bivanij.

Considering the vital roles of TFs in environmental stresses, several key TFs, such as AP2/ERF [59], AREB/ABF [62], MYB [63], WRKY [64], NAC [65] and bZIP [66] have been reported to improve drought tolerance in different plants through genetic engineering approach. The TF, *CIPK25* isolated from chickpea enhanced the root growth and improved drought and salinity stresses in tobacco [67]. Several target stress responsive genes, such as RD29A, RD10, COR15A, COR47, KIN1 and DREB2A have been reported to be induced in Arabidopsis due to the transgene *CarNAC4* isolated from chickpea [68]. The expression levels of several *CaNAC* genes have been assessed between two chickpea contrasting genotypes under dehydration stress and the TF *CaNAC16* was introduced as potential candidate gene, contributing to the better drought tolerance of Hashem and ILC482 cultivars [69]. Noteworthy, we found the gene *CaNAC16* (Ca_18090) up-regulated in Bivanijas compared to Hashem under the drought stress, indicating the vital role of this TF during plant drought tolerance.

Remarkably, five DEGs identified in this study were found in two drought tolerance-related “*QTL-hotspot”* regions in chickpea. The Ca_04561 gene encoding E3 ubiquitin-protein ligase, has been reported to be involved in the increasing of proline contents and enhancing of drought tolerance in Arabidopsis [70]. Finally, expression profiling through qRT-PCR of 12 selected genes supported the results obtained through the RNA-Seq analysis.

## Conclusion

In summary, the transcriptome analysis revealed complex genes and networks involved in chickpea drought tolerance. The gene expression values were different across the tissues, followed by the treatments and the genotypes. Although, a high number of genes were differentially expressed in the roots of both genotypes under the stress condition, however the comparison between the genotypes exhibited the important role of the shoots for conferring higher tolerance in Bivanij. In other words, the genotypes employed same genes and mechanisms in the roots as compared to the shoots. GO and KEGG pathway analysis identified several molecular mechanisms involved in the stress response and their corresponding drought-related pathways. The role of key transcription factors in the drought tolerance mechanism has been deciphered and these genes will serve as useful targets for both future research and breeding for drought tolerance/abiotic stresses in chickpea.

## Supporting information

S1 Table List of primers used in the experiment. (DOC)

S2 Table Drought responsive genes in the root tissues. (XLS)

S3 Table Drought responsive genes in the shoot tissues. (XLS)

S4 Table Drought responsive genes encoding TFs in the root tissues. (XLS)

S5 Table Drought responsive genes encoding TFs in the shoot tissues. (XLS)

S6 Table DEGs in “*QTL-hotspot*” reported in Kale et al. 2015. (DOC)

S7 Table Stress-related annotations for differentially expressed genes between the genotypes under drought stress. The genes were also annotated for some important characteristics involved in various stresses.
(DOC)

S1 Fig Number of up and down-regulated genes for 12 comparative combinations of the samples. (DOC)

S2 Fig GO analysis of 4572 differentially expressed genes in the experiment. (DOC)

S3 Fig Distribution of 20 top TF families identified in 4572 DEGs. (DOC)

S4 Fig Differentially expressed genes identified in the “*QTL-hotspot*_*a*” and “*QTL-hotspot*_*b*” in the present study. The genes corresponding to the present DEGs are indicated with the red arrows.
(DOC)

## References

[1] Food and Agriculture Organization of the United Nations, FAOSTAT. Rome, Italy. FAO; 2016. Available at: http://fao.org/faostat/en/#data/QC (Accessed 13 Jan 2018).

[2] Varshney RK, Song C, Saxena RK, Azam S, Yu S, Sharpe AG, et al Draft genome sequence of chickpea (*Cicer arietinum*) provides a resource for trait improvement. Nat Biotechnol. 2013;31(3): 240–246. doi: 10.1038/nbt.2491 23354103 10.1038/nbt.2491

[3] Jukanti AK, Gaur PM, Gowda C, Chibbar RN. Nutritional quality and health benefits of chickpea (*Cicer arietinum* L.): A review. Br J Nutr. 2012;108(S1): S11–S26. 22916806 10.1017/S0007114512000797

[4] Kashiwagi J, Krishnamurthy L, Crouch JH, Serraj R. Variability of root length density and its contributions to seed yield in chickpea (*Cicer arietinum* L.) under terminal drought stress. Field Crops Res. 2006;95(2–3): 171–181.

[5] Krishnamurthy L, Kashiwagi J, Upadhyaya H, Gowda C, Gaur P, Singh S, et al Partitioning coefficient—A trait that contributes to drought tolerance in chickpea. Field Crops Res. 2013;149: 354–365.

[6] Thudi M, Bohra A, Nayak SN, Varghese N, Shah TM, Penmetsa RV, et al Novel SSR markers from BAC-end sequences, DArT arrays and a comprehensive genetic map with 1,291 marker loci for chickpea (*Cicer arietinum* L.). PLoS ONE. 2011;6(11): e27275 doi: 10.1371/journal.pone.0027275 22102885 10.1371/journal.pone.0027275 PMC3216927

[7] Varshney RK, Kudapa H, Pazhamala L, Chitikineni A, Thudi M, Bohra A, et al Translational genomics in agriculture: some examples in grain legumes. Crit Rev Plant Sci. 2015;34(1–3): 169–194.

[8] Varshney RK, Thudi M, Nayak SN, Gaur PM, Kashiwagi J, Krishnamurthy L, et al Genetic dissection of drought tolerance in chickpea (*Cicer arietinum* L.). Theor Appl Genet. 2014;127(2): 445–462. doi: 10.1007/s00122-013-2230-6 24326458 10.1007/s00122-013-2230-6 PMC3910274

[9] Thudi M, Upadhyaya HD, Rathore A, Gaur PM, Krishnamurthy L, Roorkiwal M, et al Genetic dissection of drought and heat tolerance in chickpea through genome-wide and candidate gene-based association mapping approaches. PLoS ONE. 2014;9(5): e96758 doi: 10.1371/journal.pone.0096758 24801366 10.1371/journal.pone.0096758 PMC4011848

[10] Jaganathan D, Thudi M, Kale S, Azam S, Roorkiwal M, Gaur PM, et al Genotyping-by-sequencing based intra-specific genetic map refines a ''*QTL-hotspot*" region for drought tolerance in chickpea. Mol Genet Genomics. 2015;290(2): 559–571. doi: 10.1007/s00438-014-0932-3 25344290 10.1007/s00438-014-0932-3 PMC4361754

[11] Kale SM, Jaganathan D, Ruperao P, Chen C, Punna R, Kudapa H, et al Prioritization of candidate genes in "*QTL-hotspot*" region for drought tolerance in chickpea (*Cicer arietinum* L.). Sci Rep. 2015;5: 15296 doi: 10.1038/srep15296 26478518 10.1038/srep15296 PMC4609953

[12] Thudi M, Li Y, Jackson SA, May GD, Varshney RK. Current state-of-art of sequencing technologies for plant genomics research. Brief Funct Genomics. 2012;11(1): 3–11. doi: 10.1093/bfgp/elr045 22345601 10.1093/bfgp/elr045

[13] Michael TP, Jackson S. The first 50 plant genomes. Plant Genome. 2013; 6(2).

[14] Ohashi H, Hasegawa M, Wakimoto K, Miyamoto-Sato E. Next-generation technologies for multiomics approaches including interactome sequencing. BioMed Res Int. 2015; http://dx.doi.org/10.1155/2015/104209. 10.1155/2015/104209 PMC4306365 25649523

[15] Jain M, Misra G, Patel RK, Priya P, Jhanwar S, Khan AW, et al A draft genome sequence of the pulse crop chickpea (*Cicer arietinum* L.). Plant J. 2013;74(5): 715–729. doi: 10.1111/tpj.12173 23489434 10.1111/tpj.12173

[16] Thudi M, Khan AW, Kumar V, Gaur PM, Katta K, Garg V, et al Whole genome re-sequencing reveals genome-wide variations among parental lines of 16 mapping populations in chickpea (*Cicer arietinum* L.). BMC Plant Biol. 2016;16 Suppl 1: 10. 26822060 10.1186/s12870-015-0690-3 PMC4895712

[17] Thudi M, Chitikineni A, Liu X, He W, Roorkiwal M, Yang W, et al Recent breeding programs enhanced genetic diversity in both desi and kabuli varieties of chickpea (*Cicer arietinum* L.). Sci Rep. 2016;6: 38636 doi: 10.1038/srep38636 27982107 10.1038/srep38636 PMC5159902

[18] Hiremath PJ, Farmer A, Cannon SB, Woodward J, Kudapa H, Tuteja R, et al Large‐scale transcriptome analysis in chickpea (*Cicer arietinum* L.), an orphan legume crop of the semi‐arid tropics of Asia and Africa. Plant Biotechnol J. 2011;9(8): 922–931. doi: 10.1111/j.1467-7652.2011.00625.x 21615673 10.1111/j.1467-7652.2011.00625.x PMC3437486

[19] Kudapa H, Azam S, Sharpe AG, Taran B, Li R, Deonovic B, et al Comprehensive transcriptome assembly of Chickpea (*Cicer arietinum* L.) using sanger and next generation sequencing platforms: development and applications. PLoS ONE. 2014;9(1): e86039 doi: 10.1371/journal.pone.0086039 24465857 10.1371/journal.pone.0086039 PMC3900451

[20] Pradhan S, Bandhiwal N, Shah N, Kant C, Gaur R, Bhatia S. Global transcriptome analysis of developing chickpea (*Cicer arietinum* L.) seeds. Front Plant Sci. 2014;5: 698 doi: 10.3389/fpls.2014.00698 25566273 10.3389/fpls.2014.00698 PMC4267183

[21] Garg R, Bhattacharjee A, Jain M. Genome-scale transcriptomic insights into molecular aspects of abiotic stress responses in chickpea. Plant Mol Biol Rep. 2015;33(3): 388–400.

[22] Garg R, Shankar R, Thakkar B, Kudapa H, Krishnamurthy L, Mantri N, et al Transcriptome analyses reveal genotype-and developmental stage-specific molecular responses to drought and salinity stresses in chickpea. Sci Rep. 2016;6: 19228 doi: 10.1038/srep19228 26759178 10.1038/srep19228 PMC4725360

[23] Wang Z, Gerstein M, Snyder M. RNA-Seq: a revolutionary tool for transcriptomics. Nat Rev Genet. 2009;10(1): 57–63. doi: 10.1038/nrg2484 19015660 10.1038/nrg2484 PMC2949280

[24] Zhao S, Fung-Leung WP, Bittner A, Ngo K, Liu X. Comparison of RNA-Seq and microarray in transcriptome profiling of activated T cells. PLoS ONE. 2014;9(1): e78644 doi: 10.1371/journal.pone.0078644 24454679 10.1371/journal.pone.0078644 PMC3894192

[25] Fracasso A, Trindade LM, Amaducci S. Drought stress tolerance strategies revealed by RNA-Seq in two sorghum genotypes with contrasting WUE. BMC Plant Biol. 2016;16(1): 115 doi: 10.1186/s12870-016-0800-x 27208977 10.1186/s12870-016-0800-x PMC4875703

[26] Hubner S, Korol AB, Schmid KJ. RNA-Seq analysis identifies genes associated with differential reproductive success under drought-stress in accessions of wild barley *Hordeum spontaneum*. BMC Plant Biol. 2015;15: 134 doi: 10.1186/s12870-015-0528-z 26055625 10.1186/s12870-015-0528-z PMC4459662

[27] Bhardwaj AR, Joshi G, Kukreja B, Malik V, Arora P, Pandey R, et al Global insights into high temperature and drought stress regulated genes by RNA-Seq in economically important oilseed crop *Brassica juncea*. BMC Plant Biol. 2015; 15:9 doi: 10.1186/s12870-014-0405-1 25604693 10.1186/s12870-014-0405-1 PMC4310166

[28] Bolger AM, Lohse M, Usadel B. Trimmomatic: a flexible trimmer for Illumina sequence data. Bioinformatics. 2014;30(15): 2114–2120. doi: 10.1093/bioinformatics/btu170 24695404 10.1093/bioinformatics/btu170 PMC4103590

[29] Katta MA, Khan AW, Doddamani D, Thudi M, Varshney RK. NGS-QCbox and Raspberry for parallel automated and rapid quality control analysis of large-scale next generation sequencing (Illumina) data. PLoS ONE. 2015;10(10): e0139868 doi: 10.1371/journal.pone.0139868 26460497 10.1371/journal.pone.0139868 PMC4604202

[30] Trapnell C, Roberts A, Goff L, Pertea G, Kim D, Kelley DR, et al Differential gene and transcript expression analysis of RNA-seq experiments with TopHat and Cufflinks. Nat Protoc. 2012;7(3): 562–578. doi: 10.1038/nprot.2012.016 22383036 10.1038/nprot.2012.016 PMC3334321

[31] Trapnell C, Pachter L, Salzberg SL. TopHat: discovering splice junctions with RNA-Seq. Bioinformatics. 2009;25(9): 1105–1111. doi: 10.1093/bioinformatics/btp120 19289445 10.1093/bioinformatics/btp120 PMC2672628

[32] Trapnell C, Williams BA, Pertea G, Mortazavi A, Kwan G, Van Baren MJ, et al Transcript assembly and quantification by RNA-Seq reveals unannotated transcripts and isoform switching during cell differentiation. Nat Biotechnol. 2010;28(5): 511–515. doi: 10.1038/nbt.1621 20436464 10.1038/nbt.1621 PMC3146043

[33] Roberts A, Pimentel H, Trapnell C, Pachter L Identification of novel transcripts in annotated genomes using RNA-Seq. Bioinformatics (2011), 27: 2325–2329. doi: 10.1093/bioinformatics/btr355 21697122 10.1093/bioinformatics/btr355

[34] Trapnell C, Hendrickson DG, Sauvageau M, Goff L, Rinn JL, Pachter L. Differential analysis of gene regulation at transcript resolution with RNA-seq. Nat Biotechnol. 2013;31(1): 46–53. doi: 10.1038/nbt.2450 23222703 10.1038/nbt.2450 PMC3869392

[35] Howe EA, Sinha R, Schlauch D, Quackenbush J. RNA-Seq analysis in MeV. Bioinformatics. 2011;27(22): 3209–3210. doi: 10.1093/bioinformatics/btr490 21976420 10.1093/bioinformatics/btr490 PMC3208390

[36] Conesa A, Gotz S, Garcia-Gomez JM, Terol J, Talon M, Robles M. Blast2GO: a universal tool for annotation, visualization and analysis in functional genomics research. Bioinformatics. 2005;21(18): 3674–3676. doi: 10.1093/bioinformatics/bti610 16081474 10.1093/bioinformatics/bti610

[37] Livak KJ, Schmittgen TD. Analysis of relative gene expression data using real-time quantitative PCR and the 2^− ΔΔCT^ method. Methods. 2001;25(4): 402–408. doi: 10.1006/meth.2001.1262 11846609 10.1006/meth.2001.1262

[38] Thudi M, Gaur PM, Krishnamurthy L, Mir RR, Kudapa H, Fikre A, et al Genomics-assisted breeding for drought tolerance in chickpea. Funct Plant Biol. 2014;41(11): 1178–1190. 10.1071/FP13318 32481067

[39] Varshney RK, Hiremath PJ, Lekha P, Kashiwagi J, Balaji J, Deokar AA, et al A comprehensive resource of drought-and salinity-responsive ESTs for gene discovery and marker development in chickpea (*Cicer arietinum* L.). BMC Genomics. 2009;10(1): 1. 19912666 10.1186/1471-2164-10-523 PMC2784481

[40] Sah SK, Reddy KR, Li J. Abscisic acid and abiotic stress tolerance in crop plants. Front Plant Sci. 2016;7: 571 doi: 10.3389/fpls.2016.00571 27200044 10.3389/fpls.2016.00571 PMC4855980

[41] Jaradat MR, Feurtado JA, Huang D, Lu Y, Cutler AJ. Multiple roles of the transcription factor *AtMYBR1*/*AtMYB44* in ABA signaling, stress responses, and leaf senescence. BMC Plant Biol. 2013;13(1): 1. 24286353 10.1186/1471-2229-13-192 PMC4219380

[42] Takasaki H, Maruyama K, Takahashi F, Fujita M, Yoshida T, Nakashima K, et al *SNAC‐As*, stress‐responsive NAC transcription factors, mediate ABA‐inducible leaf senescence. Plant J. 2015;84(6): 1114–1123. doi: 10.1111/tpj.13067 26518251 10.1111/tpj.13067

[43] McAdam SA, Brodribb TJ. The evolution of mechanisms driving the stomatal response to vapor pressure deficit. Plant Physiol. 2015;167(3): 833–843. doi: 10.1104/pp.114.252940 25637454 10.1104/pp.114.252940 PMC4348763

[44] Yoshiba Y, Kiyosue T, Nakashima K, Yamaguchi-Shinozaki K, Shinozaki K. Regulation of levels of proline as an osmolyte in plants under water stress. Plant Cell Physiol. 1997;38(10): 1095–1102. 9399433 10.1093/oxfordjournals.pcp.a029093

[45] Liang X, Zhang L, Natarajan SK, Becker DF. Proline mechanisms of stress survival. Antioxid Redox Signal. 2013;19(9): 998–1011. doi: 10.1089/ars.2012.5074 23581681 10.1089/ars.2012.5074 PMC3763223

[46] Sade N, Gebremedhin A, Moshelion M. Risk-taking plants: anisohydric behavior as a stress-resistance trait. Plant Signal Behav. 2012;7(7): 767–770. doi: 10.4161/psb.20505 22751307 10.4161/psb.20505 PMC3583960

[47] Zhou Z, Ma H, Lin K, Zhao Y, Chen Y, Xiong Z, et al RNA-seq reveals complicated transcriptomic responses to drought stress in a non-model tropic plant, *Bombax ceiba* L. Evol Bioinform Online. 2015;11(Suppl 1): 27–37. doi: 10.4137/EBO.S20620 26157330 10.4137/EBO.S20620 PMC4479181

[48] Patakas A, Nikolaou N, Zioziou E, Radoglou K, Noitsakis B. The role of organic solute and ion accumulation in osmotic adjustment in drought-stressed grapevines. Plant Sci. 2002;163(2): 361–367.

[49] Ruan YL, Jin Y, Yang YJ, Li GJ, Boyer JS. Sugar input, metabolism, and signaling mediated by invertase: roles in development, yield potential, and response to drought and heat. Mol Plant. 2010;3(6): 942–955. doi: 10.1093/mp/ssq044 20729475 10.1093/mp/ssq044

[50] Lei Y, Yin C, Li C. Differences in some morphological, physiological, and biochemical responses to drought stress in two contrasting populations of *Populus przewalskii*. Physiol Plant. 2006;127(2): 182–191.

[51] Xue GP, McIntyre CL, Jenkins CL, Glassop D, van Herwaarden AF, Shorter R. Molecular dissection of variation in carbohydrate metabolism related to water-soluble carbohydrate accumulation in stems of wheat. Plant Physiol. 2008;146(2): 441–454. doi: 10.1104/pp.107.113076 18083795 10.1104/pp.107.113076 PMC2245852

[52] Din J, Khan S, Ali I, Gurmani A. Physiological and agronomic response of canola varieties to drought stress. J Anim Plant Sci. 2011;21(1): 78–82.

[53] Pourcel L, Routaboul JM, Cheynier V, Lepiniec L, Debeaujon I. Flavonoid oxidation in plants: from biochemical properties to physiological functions. Trends Plant Sci. 2007;12(1): 29–36. doi: 10.1016/j.tplants.2006.11.006 17161643 10.1016/j.tplants.2006.11.006

[54] Wang F, Kong W, Wong G, Fu L, Peng R, Li Z, et al AtMYB12 regulates flavonoids accumulation and abiotic stress tolerance in transgenic *Arabidopsis thaliana*. Mol Genet Genomics. 2016: 1–15. 10.1007/s00438-016-1203-2 27033553

[55] Shao HB, Chu LY, Lu ZH, Kang CM. Primary antioxidant free radical scavenging and redox signaling pathways in higher plant cells. Int J Biol Sci. 2008;4(1): 8. 10.7150/ijbs.4.8 PMC2140154 18167531

[56] Agarwal P, Jha B. Transcription factors in plants and ABA dependent and independent abiotic stress signaling. Biol Plant. 2010;54(2): 201–212.

[57] Bhargava S, Sawant K. Drought stress adaptation: metabolic adjustment and regulation of gene expression. Plant Breeding. 2013;132(1): 21–32.

[58] Nakashima K, Yamaguchi-Shinozaki K, Shinozaki K. The transcriptional regulatory network in the drought response and its crosstalk in abiotic stress responses including drought, cold, and heat. Front Plant Sci. 2014;5: 170 doi: 10.3389/fpls.2014.00170 24904597 10.3389/fpls.2014.00170 PMC4032904

[59] Xu ZS, Chen M, Li LC, Ma YZ. Functions and application of the AP2/ERF transcription factor family in crop improvement. J Integr Plant Biol. 2011;53(7): 570–85. doi: 10.1111/j.1744-7909.2011.01062.x 21676172 10.1111/j.1744-7909.2011.01062.x

[60] Xu ZS, Li ZY, Chen Y, Chen M, Li LC, Ma YZ. Heat shock protein 90 in plants: molecular mechanisms and roles in stress responses. Int J Mol Sci. 2012;13(12): 15706–23. doi: 10.3390/ijms131215706 23443089 10.3390/ijms131215706 PMC3546657

[61] Agarwal G, Garg V, Kudapa H, Doddamani D, Pazhamala LT, Khan AW, et al Genome-wide dissection of AP2/ERF and HSP90 gene families in five legumes and expression profiles in chickpea and pigeonpea. Plant Biotechnol J. 2016;14(7): 1563–1577. doi: 10.1111/pbi.12520 26800652 10.1111/pbi.12520 PMC5066796

[62] Yoshida T, Fujita Y, Maruyama K, Mogami J, Todaka D, Shinozaki K, et al Four Arabidopsis AREB/ABF transcription factors function predominantly in gene expression downstream of SnRK2 kinases in abscisic acid signaling in response to osmotic stress. Plant Cell Environ. 2015;38(1): 35–49. doi: 10.1111/pce.12351 24738645 10.1111/pce.12351 PMC4302978

[63] Su LT, Li JW, Liu DQ, Zhai Y, Zhang HJ, Li XW, et al A novel MYB transcription factor, GmMYBJ1, from soybean confers drought and cold tolerance in *Arabidopsis thaliana*. Gene. 2014;538(1): 46–55. doi: 10.1016/j.gene.2014.01.024 24440241 10.1016/j.gene.2014.01.024

[64] Liu L, Zhang Z, Dong J, Wang T. Overexpression of MtWRKY76 increases both salt and drought tolerance in *Medicago truncatula*. Environ Exper Bot. 2016; 123:50–58.

[65] Nakashima K, Tran LSP, Van Nguyen D, Fujita M, Maruyama K, Todaka D, et al Functional analysis of a NAC‐type transcription factor OsNAC6 involved in abiotic and biotic stress‐responsive gene expression in rice. Plant J. 2007;51(4): 617–630. doi: 10.1111/j.1365-313X.2007.03168.x 17587305 10.1111/j.1365-313X.2007.03168.x

[66] Hsieh TH, Li CW, Su RC, Cheng CP, Tsai YC, Chan MT. A tomato bZIP transcription factor, SlAREB, is involved in water deficit and salt stress response. Planta. 2010;231(6): 1459–1473. doi: 10.1007/s00425-010-1147-4 20358223 10.1007/s00425-010-1147-4

[67] Meena MK, Ghawana S, Dwivedi V, Roy A, Chattopadhyay D. Expression of chickpea CIPK25 enhances root growth and tolerance to dehydration and salt stress in transgenic tobacco. Front Plant Sci. 2015;6: 683 doi: 10.3389/fpls.2015.00683 26442004 10.3389/fpls.2015.00683 PMC4561800

[68] Yu X, Liu Y, Wang S, Tao Y, Wang Z, Shu Y, et al CarNAC4, a NAC-type chickpea transcription factor conferring enhanced drought and salt stress tolerances in Arabidopsis. Plant Cell Rep. 2016;35(3): 613–627. doi: 10.1007/s00299-015-1907-5 26650836 10.1007/s00299-015-1907-5

[69] Nguyen KH, Ha CV, Watanabe Y, Tran UT, Nasr Esfahani M, Nguyen DV, et al Correlation between differential drought tolerability of two contrasting drought-responsive chickpea cultivars and differential expression of a subset of CaNAC genes under normal and dehydration conditions. Front Plant Sci. 2015; 6:449 doi: 10.3389/fpls.2015.00449 26150822 10.3389/fpls.2015.00449 PMC4472984

[70] Ju HW, Min JH, Chung MS, Kim CS. The atrzf1 mutation of the novel RING-type E3 ubiquitin ligase increases proline contents and enhances drought tolerance in Arabidopsis. Plant Sci. 2013;203: 1–7. doi: 10.1016/j.plantsci.2012.12.007 23415322 10.1016/j.plantsci.2012.12.007

