# Expression of Concern: RNA-Seq analysis revealed genes associated with drought stress response in kabuli chickpea (*Cicer arietinum* L.)

**DOI:** 10.1371/journal.pone.0350349

**Published:** 2026-06-01

**Authors:** The PLOS One Editors

The *PLOS One* Editors issue this Expression of Concern because this article [1] is one of a series of articles for which there are concerns about competing interests and the reliability of peer review. The competing interests were not disclosed as required by PLOS policy.

PLOS regrets that the peer review concerns were not identified and addressed before the article was published.

In following up on this matter, PLOS did not evaluate the article’s integrity or scientific validity.
