# Supplementary material for: RNA-Seq analysis revealed genes associated with drought stress response in kabuli chickpea (Cicer arietinum L.)
Source: PLoS One. 2018 Jun 28;13(6):e0199774. doi: 10.1371/journal.pone.0199774 (PMC6023194; doi:10.1371/journal.pone.0199774)
Supplement: S1 Table — (DOC) [file pone.0199774.s001.doc]

**S1 Table:** **List of primers used in the experiment.**

| **S. No.** | **Gene ID** | **Forward primer sequence** | **Reverse primer sequence** |
| --- | --- | --- | --- |
| 1 | Ca_00474 | CACCGTAGGCTATTCGAAGC | CGGCGGAAGTAGAACAAGAG |
| 2 | Ca_04355 | TCAGGTGCACATCTCAATCC | TTGCAAAAGCTTGACCAATG |
| 3 | Ca_04358 | GAATCACAAATGGGAGCAGCA | CTTCACAAGGCTTTGCAACAA |
| 4 | Ca_04370 | CACCGACTCATGATGGAT | TCATCTTCACGCCCTCTT |
| 5 | Ca_04561 | AATGCTTTCAAGGCCAAA | CAACCTGTTTCCACGCAA |
| 6 | Ca_16138 | TTGCATGGTTTGGTTCTTGA | TGACTTTGCTATTCCCACCC |
| 7 | Ca_04816 | CTCGCCCCTTGAAGTAATTG | TTTGCAGGTGCAGTGATGAT |
| 8 | Ca_23092 | AGCAAGAGAAGGACCATTCG | TCCTTTGGTCCCAAAAACAG |
| 9 | Ca_00047 | GCAAGCACACCAGCTTTGTA | CAACTTTGCCAGCACAAGAA |
| 10 | Ca_15236 | AAGGCACCAAAACTGATTGG | GGCTTGTTGCTGTTGTCAGA |
| 11 | Ca_11540 | AAGAACAAGGGCATTGATGG | TGCGACCTTAACGAAATCCT |
| 12 | Ca_20991 | TGCTCTCCAGCAGTGTACCA | TTTGGGTTTGATCCTCTTGG |
