# Supplementary material for: RNA-Seq analysis revealed genes associated with drought stress response in kabuli chickpea (Cicer arietinum L.)
Source: PLoS One. 2018 Jun 28;13(6):e0199774. doi: 10.1371/journal.pone.0199774 (PMC6023194; doi:10.1371/journal.pone.0199774)
Supplement: S6 Table — (DOC) [file pone.0199774.s006.doc]

**S6 Table.** **DEGs in “*QTL-hotspot*” reported in Kale et al. 2015.**

| Genomic region | Gene ID | BRCT vs BRDS | BRCT vs BSCT | BRDS vs BSDS | BSDS vs HSDS | HRCT vs HSCT | HRDS vs HSDS | HSCT vs HSDS | Annotation |
| --- | --- | --- | --- | --- | --- | --- | --- | --- | --- |
|  |  |  |  |  |  |  |  |  |  |
| “*QTL-hotspot_a*” | Ca_04551 |  | Down | Down | Up |  |  | Up | - Aldo/keto reductase/potassium channel subunit beta |
|  | Ca_04552 |  | Up | Up |  | Up | Up |  | - Chlorophyll A-B binding protein |
| “*QTL-hotspot_b*” | Ca_04561 |  | Up | Up |  | Up | Up |  | - E3 ubiquitin-protein ligase Msl2, zinc RING finger |
|  | Ca_04564 |  |  |  | Up | Up |  |  | - Leucine-rich repeat |
|  | Ca_04569 | Up |  |  |  |  |  |  | - Inositol polyphosphate-related phosphatase |
