# Supplementary material for: RNA-Seq analysis revealed genes associated with drought stress response in kabuli chickpea (Cicer arietinum L.)
Source: PLoS One. 2018 Jun 28;13(6):e0199774. doi: 10.1371/journal.pone.0199774 (PMC6023194; doi:10.1371/journal.pone.0199774)
Supplement: S7 Table — The genes were also annotated for some important characteristics involved in various stresses. (DOC) [file pone.0199774.s007.doc]

**S7** **Table:** **Stress-related annotations for differentially expressed genes between the genotypes under drought stress**

| Gene ID | Position | Tissue | Genotype | TF | Annotation |
| --- | --- | --- | --- | --- | --- |
| Ca_06635 | Ca7 | Root | Bivanij | C3H | Response to water deprivation, response to salt stress, regulation of stomatal closure |
| Ca_10437 | Ca6 | Root | Bivanij | C3H | Response to stress |
| Ca_10320 | Ca6 | Root | Hashem | - | Defense response |
| Ca_03175 | Ca7 | Root | Hashem | M-type | Response to oxidative stress, hydrogen peroxide catabolic process |
| Ca_14775,  Ca_14777 | Ca1 | Shoot | Bivanij | - | Response to biotic stimulus, defense response |
| Ca_14776 | Ca1 | Shoot | Bivanij | - | Response to biotic stimulus, defense response |
| Ca_09533 | Ca3 | Shoot | Bivanij | NAC | Defense response, Flavonoid biosynthetic process |
| Ca_03757 | Ca4 | Shoot | Bivanij | - | Defense response to bacterium, defense response to fungus |
| Ca_02982,  Ca_02983 | Ca7 | Shoot | Bivanij | - | Response to biotic stimulus, defense response |
| Ca_02986 | Ca7 | Shoot | Bivanij | - | Response to biotic stimulus, defense response |
| Ca_09920 | Ca7 | Shoot | Bivanij | - | Defense response, Chitin catabolic process |
| Ca_15236 | Ca2 | Shoot | Bivanij | NAC | Systemic acquired resistance (SAR), response to endoplasmic reticulum stress, hyperosmotic salinity response, negative regulation of leaf senescence, proline biosynthetic process, trehalose biosynthetic process |
| Ca_08560 | Ca6 | Shoot | Bivanij | - | Response to stress |
| Ca_04125 | Ca5 | Shoot | Bivanij | MYB-related | Response to oxidative stress, hydrogen peroxide catabolic process |
| Ca_14524 | Ca7 | Shoot | Bivanij | - | Response to stimulus |
| Ca_25214 | Scaffold1964 | Shoot | Bivanij | - | Response to salt stress, Response to ethylene |
| Ca_01935 | Ca8 | Shoot | Hashem | M-type | Response to oxidative stress, hydrogen peroxide catabolic process |
| Ca_12471 | Ca2 | Shoot | Hashem | - | Response to freezing |
| Ca_25602 | Scaffold3422 | Shoot | Hashem | G2-like | Response to stress |
| XLOC_029416 | Scaffold3422 | Shoot | Hashem | - | Response to stress |
| Ca_07985 | Ca1 | Shoot | Hashem | MYB-related | Response to stress, hydrogen peroxide catabolic process |
