# Supplementary figures and images for: RNA-Seq analysis revealed genes associated with drought stress response in kabuli chickpea (Cicer arietinum L.)
Source: PLoS One. 2018 Jun 28;13(6):e0199774. doi: 10.1371/journal.pone.0199774 (PMC6023194; doi:10.1371/journal.pone.0199774)

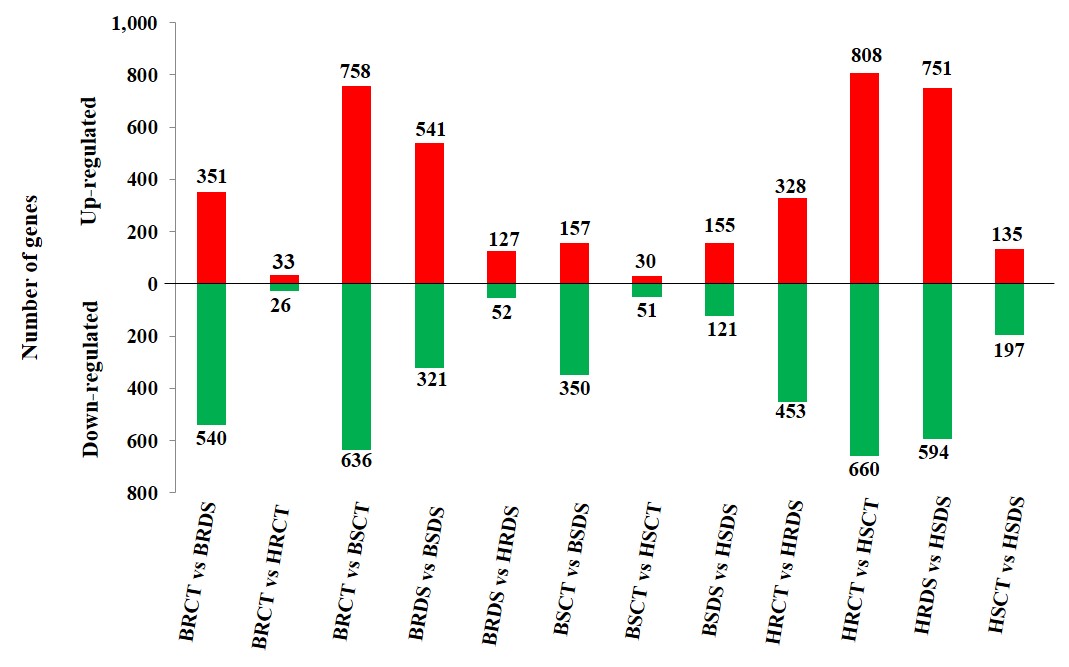


**S1 Fig.** **Number of up and down-regulated genes for 12 comparative combinations of the samples.**

Supplement: S1 Fig — (DOC) [file pone.0199774.s008.doc]

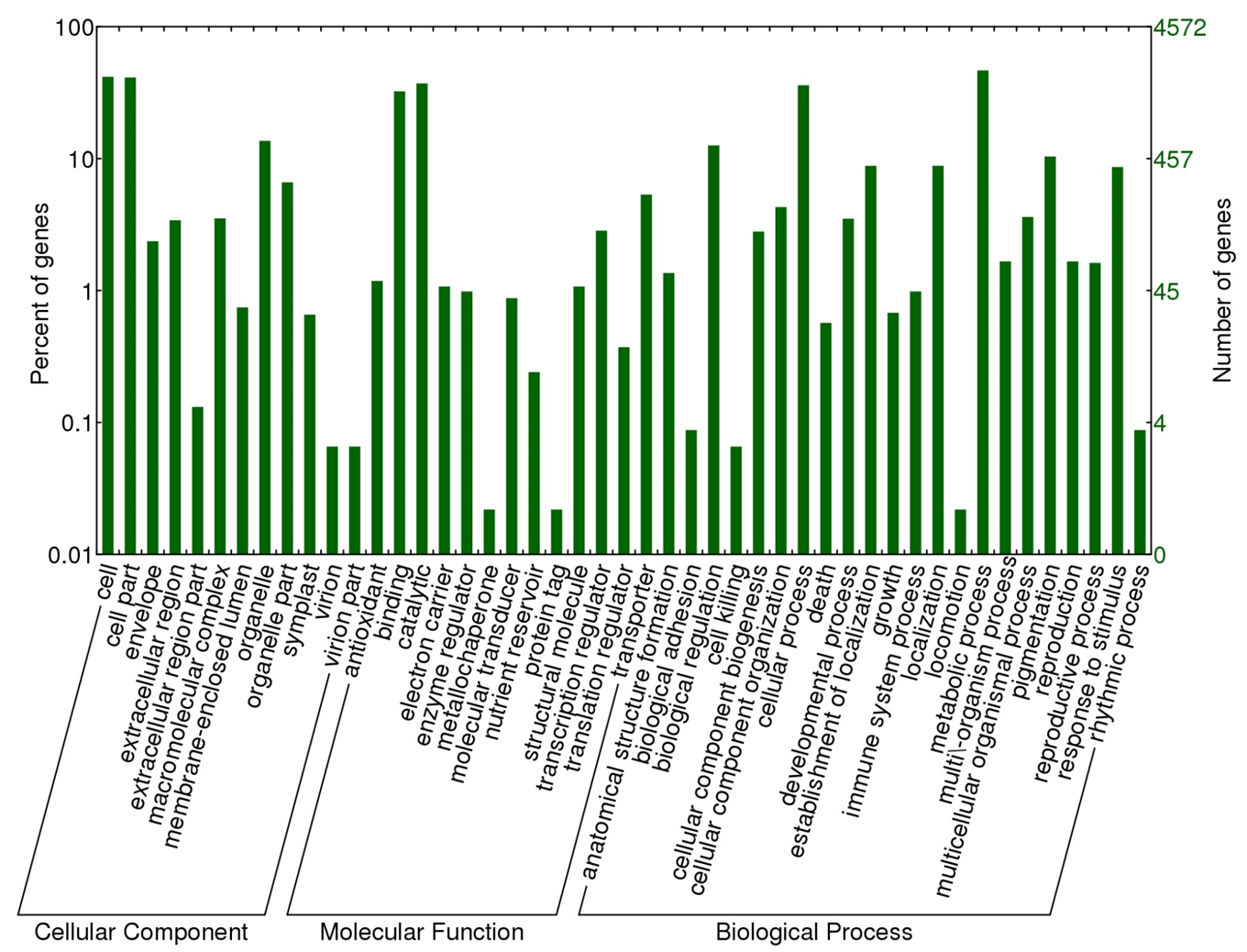


**S2 Fig.** **GO analysis for 4,572 differentially expressed genes in the experiment.**

Supplement: S2 Fig — (DOC) [file pone.0199774.s009.doc]

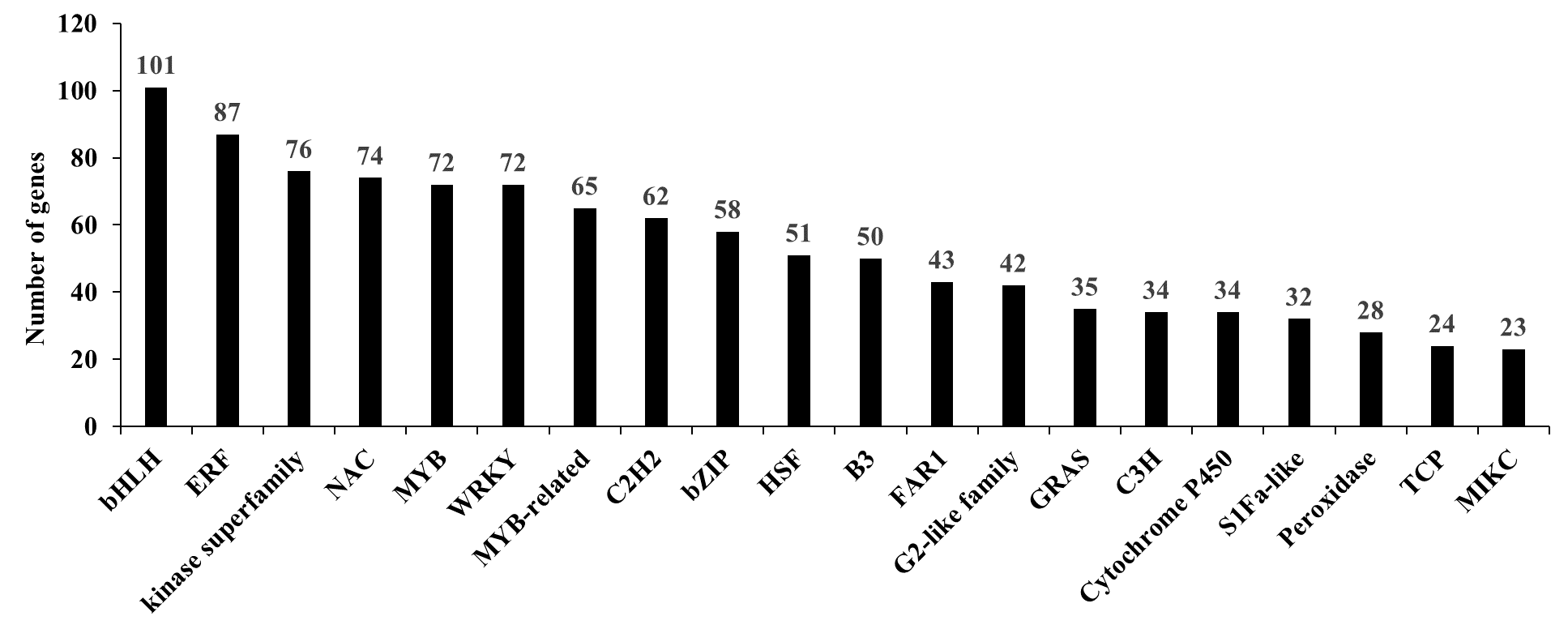


**S3 Fig. Distribution of 20 top TF families identified in the 4572 DEGs.**

Supplement: S3 Fig — (DOC) [file pone.0199774.s010.doc]
