# Supplementary material for: RNA-Seq analysis revealed genes associated with drought stress response in kabuli chickpea (Cicer arietinum L.)
Source: PLoS One. 2018 Jun 28;13(6):e0199774. doi: 10.1371/journal.pone.0199774 (PMC6023194; doi:10.1371/journal.pone.0199774)
Supplement: S4 Fig — The genes corresponding to the present DEGs are indicated with the red arrows. (DOC) [file pone.0199774.s011.doc]

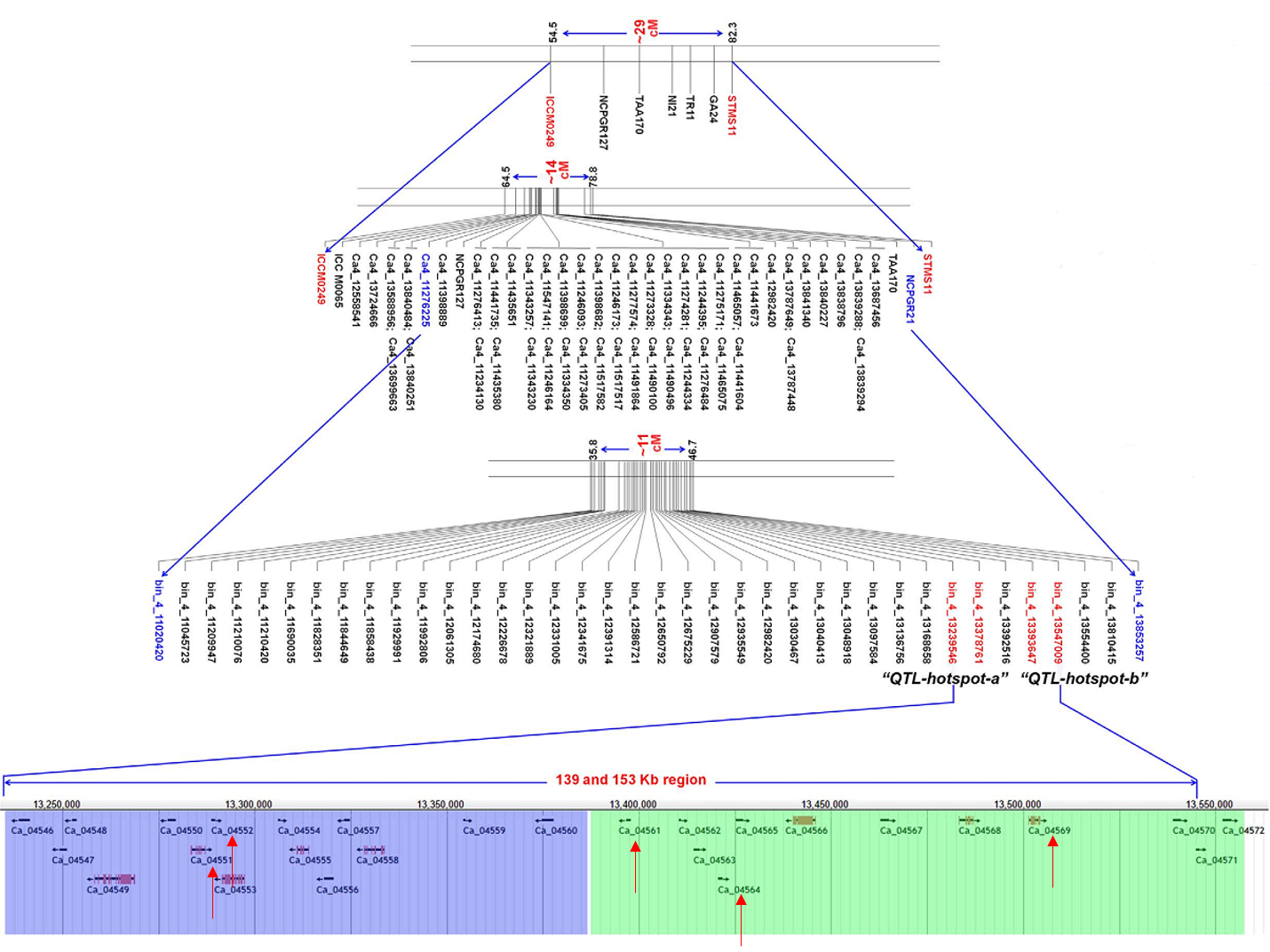


**S4 Fig. Two QTL-hotspot regions for drought tolerance in chickpea adapted from Kale et al. 2015.** The genes corresponding to the present DEGs are indicated with red arrow.
